# Neuraminidase inhibitors sensitivity in Cambodian H5N1 and H1N1 pandemic influenza viruses

**DOI:** 10.1186/1753-6561-5-S1-P68

**Published:** 2011-01-10

**Authors:** M Naughtin, S Mardy, S San, JC Dyason, M von Itztein, P Buchy

**Affiliations:** 1 Institut Pasteur in Cambodia, Phnom Penh, Cambodia; 2 Institute for Glycomics, Gold Coast Campus, Griffith University, Southport, Queensland 4222, Australia

## Background

Since 2003, H5N1 avian influenza strains have become endemic in many countries in Southeast Asia, including Cambodia. In Cambodia alone there have been 26 outbreaks in poultry flocks and 10 human cases with 9 deaths. We have analyzed the genome of a large panel of H5N1 strains isolated from poultry and human between 2004-2010. Several strains have molecular alterations which are predicted to affect sensitivity to neuraminidase inhibitors (NAI), the primary drugs of choice in the treatment of H5N1 infections. In June 2009 the first H1N1 pandemic (H1N1pdm) viruses were detected in Cambodia and have since been the main influenza strains circulating during the epidemic season. The aim of this study was primarily the surveillance of oseltamivir and zanamivir drug resistance in Cambodian H5N1 and H1N1pdm isolates.

## Methods

A chemiluminescence-based *in vitro* assay of neuraminidase (NA) activity, which utilizes the artificial NA substrate 1,2-dioxetane derivative of sialic acid (NA-STAR, Applied Biosystems^®^), was used to determine the concentration of drug required to inhibit 50% of NA enzyme activity (IC_50_).

## Results

We have identified a small number of H5N1 outliers with reduced susceptibility to NAIs and have further characterized mutations predicted to affect drug resistance using computer modeling, and recombinant viruses containing these mutations generated by reverse genetics. We have also investigated several previously described resistance mutations in the context of the Cambodian H5N1 virus using reverse genetics. We found no evidence of NAI drug resistance in H1N1pdm viruses in Cambodia.

## Conclusion

We have monitored NAI sensitivity of H5N1 and H1N1pdm viruses in Cambodia and in general have not found NAI resistant viruses with classical resistance mutations. However, we have identified naturally occurring mutations in H5N1 viruses which reduce the sensitivity to NAIs. The ongoing surveillance and understanding of novel drug resistance mutations is of great importance in the global efforts against influenza disease and pandemics.

